# Equivalent outcomes of ACL revision with over-the-top single and double-bundle reconstruction using hamstring tendon compared to anatomical single and double-bundle reconstruction

**DOI:** 10.1186/s40634-022-00472-0

**Published:** 2022-04-13

**Authors:** Goki Kamei, Atsuo Nakamae, Masakazu Ishikawa, Kyohei Nakata, Akinori Nekomoto, Shunya Tsuji, Naofumi Hashiguchi, Nobuo Adachi

**Affiliations:** 1 Department of Orthopaedic Surgery, Graduate School of Biomedical and Health Sciences, Hiroshima University, 1-2-3, Kasumi, Minami-ku, Hiroshima, Japan; 2 Department of Artificial Joints and Biomaterials, Graduate School of Biomedical and Health Sciences, Hiroshima University, Hiroshima, Japan

## Abstract

**Purpose:**

In revision anterior cruciate ligament reconstruction (ACLR), our procedure of choice is the over-the-top route (OTTR) for cases where it is difficult to use a primary bone tunnel or to create a new bone tunnel due to the enlargement or malposition of the bone tunnel. Depending on the condition of the primary bone tunnel, we choose single (bone tunnel or OTTR) or double (bone tunnel or anteromedial (AM) bundle: OTTR /posterolateral (PL) bundle: bone tunnel) for femoral fixation. This study showed the results of single and double OTTR revision ACLR using the hamstring tendon.

**Methods:**

Seventy-eight patients, who underwent revision ACLR using the hamstring tendon and who could be followed up for more than 2 year, were included in this study. The methods of revision ACLR were single in 54 cases (bone tunnel: 24 cases; OTTR method: 30 cases) and double in 24 cases (bone tunnel: 16 cases; OTTR for AM bundle and bone tunnel for PL bundle: eight cases). The cause of re-injury, the meniscus and cartilage injury, the reconstruction method, and the Lysholm score, Lachman test, Pivot-shift test, and the side-to-side difference in the tibial anterior translation were evaluated before and after surgery.

**Results:**

There was no statistically significant difference in the Lyshom score, Lachman / Pivotshift test and side-to-side difference anterior translation of the tibia between the four groups.

**Conclusions:**

The clinical results of single and double OTTR revision ACLR are equivalent to those of anatomic single and double bone tunnel procedures.

## Background

Revision anterior cruciate ligament reconstruction (ACLR) tends to be more complicated and is more difficult to treat than primary ACLR, because the technique was restricted by the harvested tendon and the location of the bone tunnel in primary surgery [3, 5, 15]. In particular, in cases where the bone tunnel cannot be created in the anatomical position due to postoperative bone tunnel enlargement or malposition of the primary bone tunnel, the choice of the surgical method is often particularly difficult [7, 13, 17].

Our graft of preference is the hamstring tendon, which use the primary bone tunnel in a single-stage procedure whenever possible. The over-the-top route (OTTR) is the method of choice for the femur, when it is difficult to use a primary bone tunnel or to create a new bone tunnel due to the enlargement of the bone tunnel [18]. The revision of OTTR ACLR has been reported to have comparable results to anatomical reconstruction, and the cadaver study has demonstrated that it is as stable as anatomical single reconstruction, making OTTR a useful method for revision ACLR [7, 8, 14, 17].

Depending on the location of the primary bone tunnel, our preferences are single bone tunnel, single OTTR, double bone tunnel, and double OTTR (AM OTTR / PL bone tunnel).

Our study presents the results of single and double OTTR revision ACLR using the hamstring tendon as well as a comparison of the different techniques (different femoral fixation methods). This study aimed to determine whether single and double OTTR revision ACLR can achieve comparable results to anatomical revision ACLR using hamstring tendon.

## Materials and methods

Ethical approval of this study was obtained from Ethical Committee for Epidemiology of Hiroshima University.

### Patient selection

**Fig. 1:**
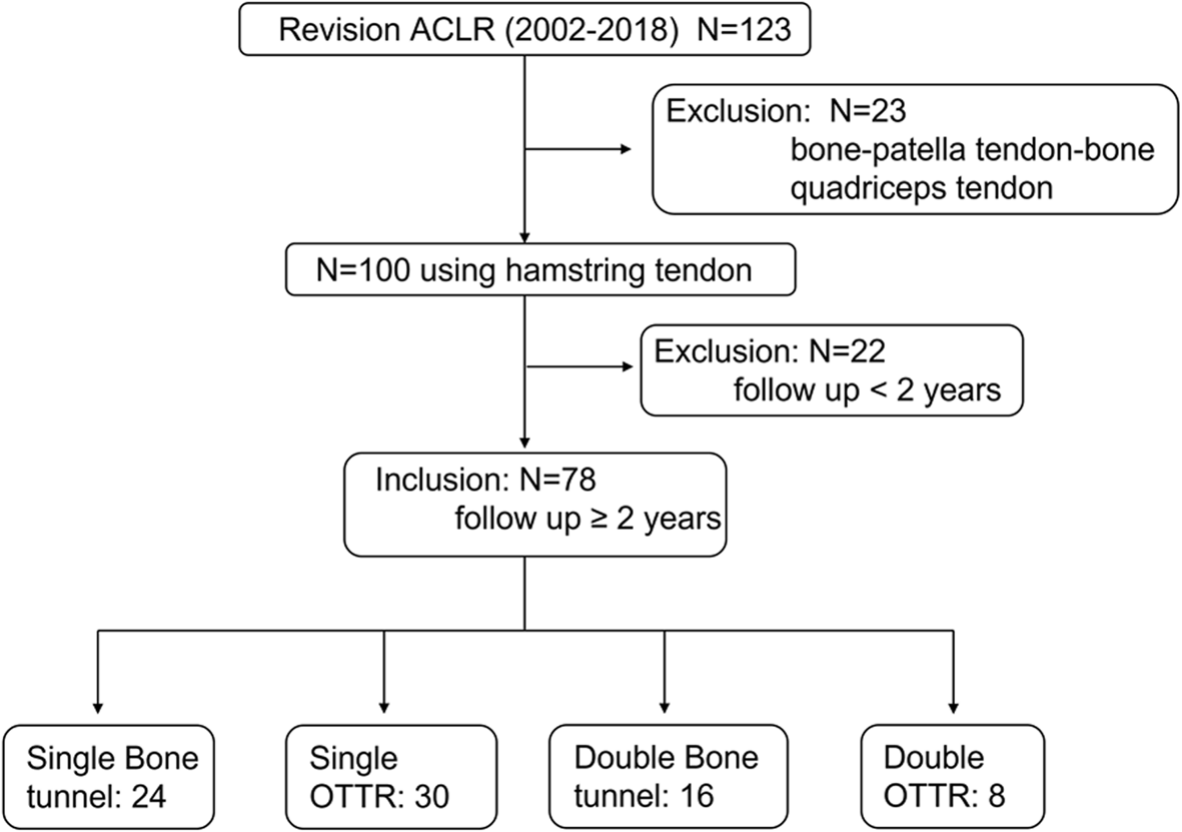
CONSORT Flow Diagram

Ninety-two of 123 patients who underwent revision ACLR between 2002 and 2018 were enrolled in this study. This retrospective study included 78 patients who underwent revision ACLR using the hamstring tendon and those who were followed up for more than 2 years. Exclusion criteria were (1) Graft: bone-patella tendon-bone and quadriceps tendon; (2) Patients could not be followed for 2 years. In cases which the initial bone tunnel was optimally positioned and there was no bone tunnel enlargement, or when a new bone tunnel could be created in the footprint by malpositioning, the bone tunnel technique (single or double) was performed. OTTR was selected for cases in which a initial bone tunnel cannot be used due to enlargement, and double OTTR was selected for cases in which an initial PL tunnel can be used or a new PL tunnel can be created. Inclusion and exclusion criteria were shown in Fig. 1. The data of patients was shown in Tables 1 and 2.

**Table 1:** Data of all included patients

All patient data	
Age	29.5 ± 10.2(16-48)
Gender (male/female)	31/47
Follow-up period (year)	3.7 ± 2.5(2-14)
Primary reconstructed method	Single 72 cases
	Double 6 cases
Graft of primary ACLR	Hamstring: 46 cases
	Artificial ligament: 20 cases
	Bone-patella tendon –bone: 5 cases
	Quadriceps tendon: 4 cases
	Iliotibial band: 3 cases
Causes of failure	Malposition of bone tunnel: 40 cases
	Artificial ligament: 20 cases
	Reinjury: 18 cases
Interval from primary reconstruction to failure (year)	7.7 ± 7.0 (0.5-28)
Interval from primary ACL failure to revision ACL (year)	2.9 ± 5.4 (0.08-17)

**Table 2:** Patient characteristics

Patient data	Single		Double	
	Bone tunnel	OTTR	Bone tunnel	AM:OTTR PL:Bone tunnel
Age	30.0 ± 10.8	30.1 ± 10.6	29.8 ± 8.89	25.3 ± 10.3
Gender (Male/Female)	8/16	13/17	8/8	2/6
Interval from primary ACLR to failure	10.0 ± 8.0	7.4 ± 7.2	6.6 ± 5.5	4.0 ± 3.9
Interval from primary ACL failure to revision ACL	2.5 ± 4.6	1.9 ± 3.4	3.2 ± 4.6	5.5 ± 12.1
Follow-up period (year)	4.1 ± 3.3	3.8 ± 2.2	3.0 ± 1.2	3.5 ± 2.8
Meniscus injury (medial/lateral/both)	9/2/3	10/4/3	5/2/4	2/0/0

### Surgical technique

**Fig. 2:**
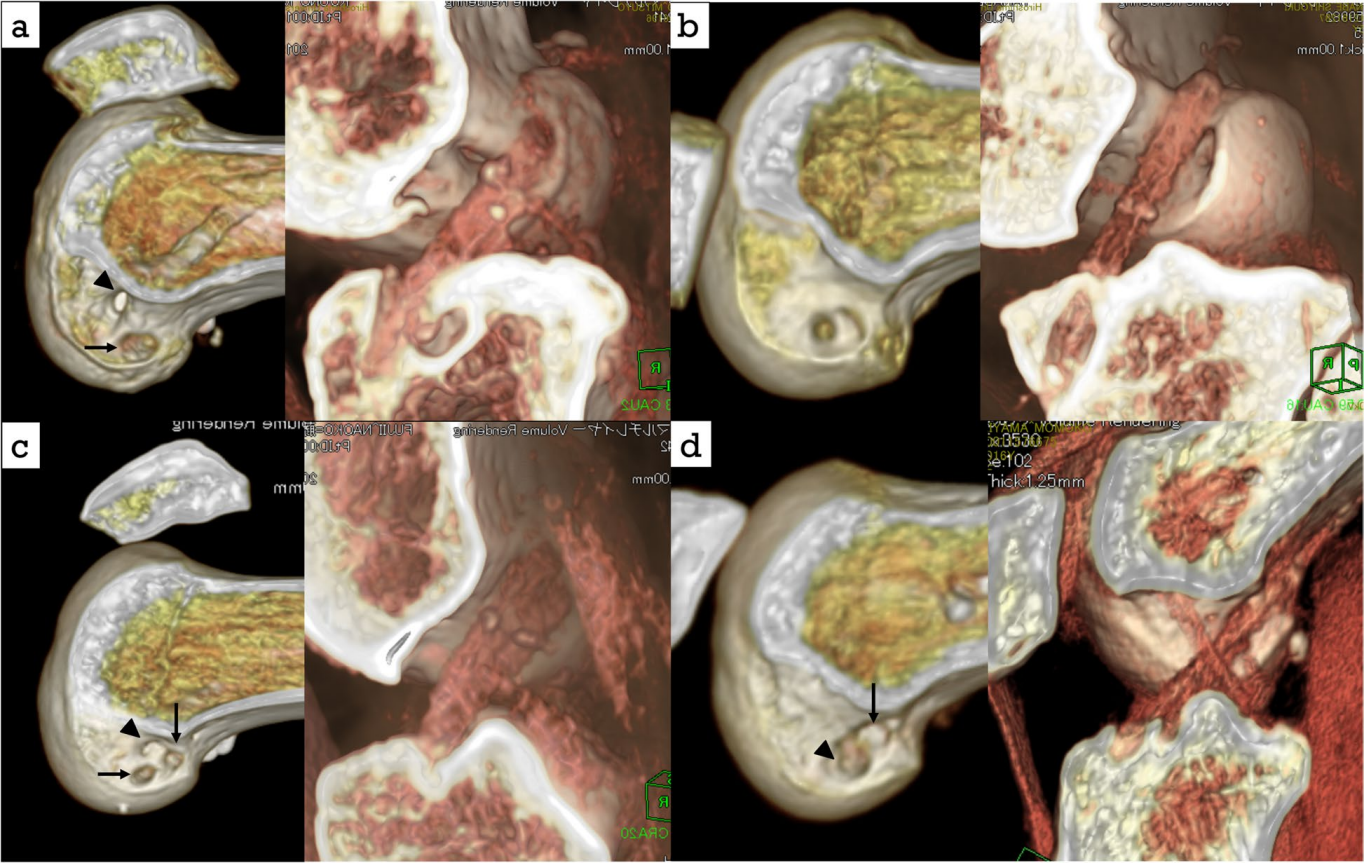
Operative procedure. **a**, Single (Mal-position): A primary bone tunnel (arrow head) was mal-positioned and a new single bone tunnel (arrow) was created. **b**, Single OTTR (Enlargement of primary bone tunnel):A new bone tunnel could not be created due to enlargement and the OTTR procedure was chosen. **c**, Single-Double (Mal-position):A primary bone tunnel (arrow head) was mal-positioned and new bone tunnels (arrow) of AM and PL bundle were created. **d**, Single-Double / AM OTTR (Re-injury case); We chose to use a primary tunnel (arrow head) for PL bundle. OTTR was selected for AM bundle because there was a risk of coalition when a primary AM tunnel (arrow) was used. The femoral side was fixed with a staple in the OTTR procedure and with Endo-Button CL in the bone tunnel procedure, and the tibial side was fixed with a staple in all cases

**Fig. 3:**
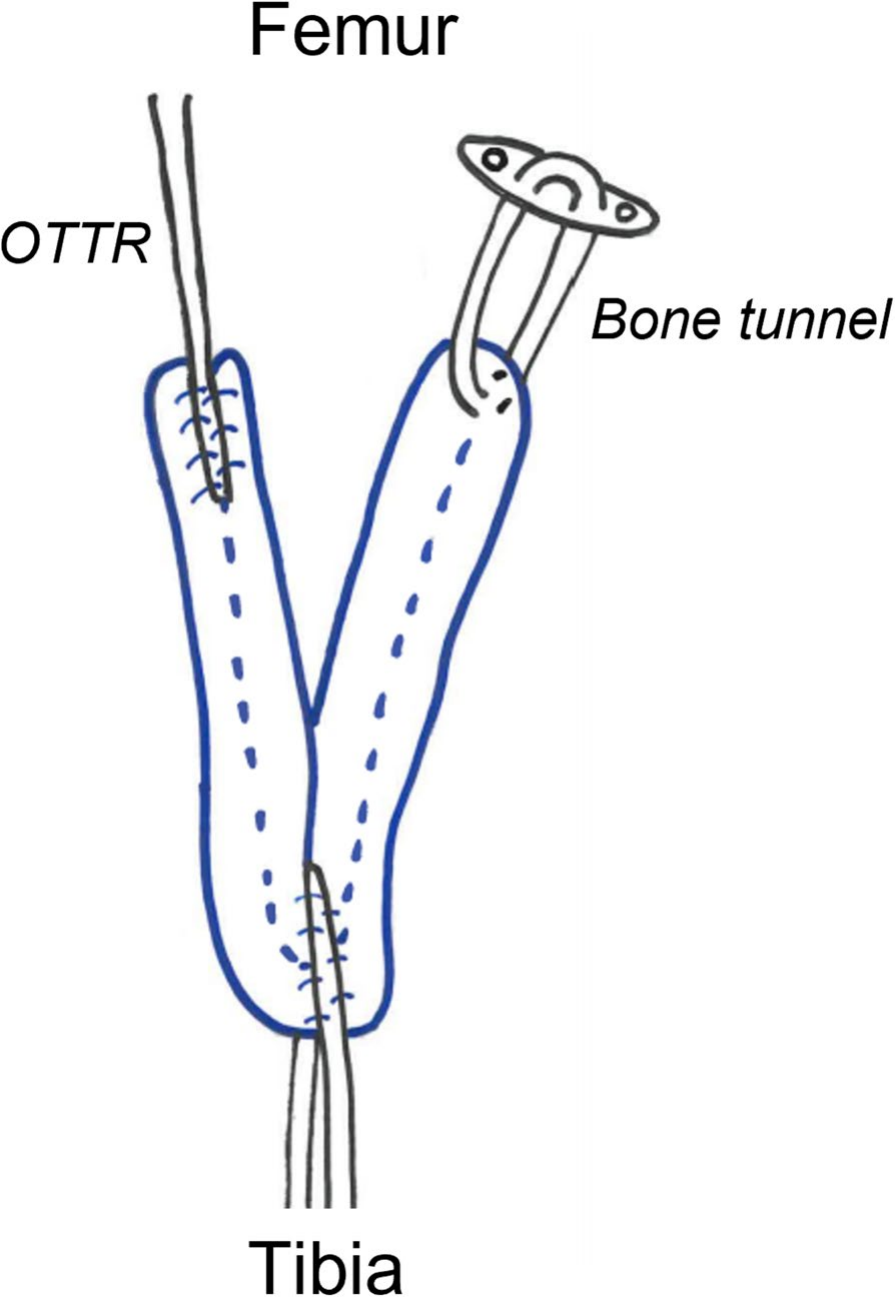
The graft for double bundle reconstruction

The methods of revision ACLR were single in 54 cases [bone tunnel method (SB group): 24 cases, OTTR method (SO group): 30 cases], femoral side double and tibial side single in 24 cases [bone tunnel method (DB group): 16 cases, OTTR for AM bundle and bone tunnel method for PL bundle (DO group): eight cases] (Fig. 2). Single revision ACLR was performed in the same way described by Usman et al. [17]. In double bundle revision ACLR, the semitendinosus tendon was folded in half through an EndoButton-CL (Smith&Nephew, Andover, MA) and the free ends were sewn with an EndoButton tape (Smith&Nephew, Andover, MA). An EndoButton tape side was used for OTTR graft and achieved with two staples. An EndoButton-CL side was used for bone tunnel graft. The graft was then folded in half through an EndoButton tape, and this EndoButton tape side was used for tibial graft, which was achieved with two staples with the tension of 50 N (Fig. 3).

### Clinical evaluation

We evaluated Lysholm score, Lachman test (positive or negative), Pivot-shift test (positive or negative), and the side-to-side difference (SSD) in tibial anterior translation using Kneelax3 (30lbs), both before and after surgery at 2 years. Postoperative failure and postoperative sports activities were evaluated. Postoperative sporting activities could be investigated in 18 cases of SB, 15 cases of SO, 12 cases of DB and seven cases of DO group. Sports activity level was at the recreational level, except for one case of DB (professional football player).

### Statistical analysis

Data was analyzed using Stat-view 5.0 (SAS Institute Inc., Vary, NC, USA). Results were expressed as the mean ± standard deviation. Statistical analysis between four groups was performed with a one-way Analysis of Variance and the Chi-squared test. Post hoc test was performed with Mann-Whitney U-test with Bonferroni correction. *P* < 0.05 was considered statistically significant.

## Results

The data of clinical results are shown in Table 3.

**Table 3:** A comparison of post-operative results (Lysholm score, Lachman test, Pivot-shift test and side-to-side difference in anterior translation of tibia) among the four groups

Clinical results	Single		Double	
	Bone tunnel	OTTR	Bone tunnel	AM: OTTR PL: Bone tunnle
Lysholm score	93.9 ± 7.4	94.3 ± 6.0	96.7 ± 4.7	95.6 ± 5.6
Lachman test (positive/negative)	2/22	1/29	1/15	1/7
Pivot-shift test (positive/negative)	4/20	4/26	2/14	1/7
Side-to-side difference in Anterior Translation of tibia using kneelax 3 at 30lbs (mm)	1.44 ± 3.2	0.73 ± 2.1	1.54 ± 2.7	0.53 ± 2.0

### Laxity

There was no statistically significant difference in the Lachman and Pivot-shift test in any of the four groups. The mean SSD in the anterior translation of the tibia was 1.44 ± 3.2 mm in SB, 0.73 ± 2.1 mm in SO, 1.54 ± 2.7 mm in DB and 0.53 ± 2.0 mm in DO groups. There was no statistically significant difference in SSD in anterior translation of the tibia in any of the four groups. Failure of over 5 mm was observed in two cases in the SB and one case in DB groups; failure was defined based on von Essen et al. [20].

### Lysholm score

The mean Lysholm score was 93.9 ± 7.4 in SB, 94.3 ± 6.0 in SO, 96.7 ± 4.7 in DB and 95.6 ± 5.6 mm in DO groups. There was no statistically significant difference in Lysholm score in any of the four groups.

None of the patients had a poor prognosis. Fair cases were found in two in SB group and one in SO. Ten good cases were found in the SB group, 12 in SO, four in DB and two in DO groups. Twelve excellent cases were found in SB, 17 in SO, 12 in DB and six in DO groups. (Lysholm score; poor:< 65, Fair: 65-83, Good: 84-90, Excellent: > 90).

### Postoperative complications and daily activity

OA progression was observed in one case in SB and two cases in SO group. Meniscus injury was occurred in three cases in SB (medial meniscus: one case, lateral meniscus: two cases) and two cases (medial meniscus: two cases) in SO group. A partial meniscectomy was performed in all the paitents with meniscus injury. Contralateral ACL injury was occurred in 1 case in SB group and ACL reconstruction was performed. Reinjury was occurred in 1 case in SO, DB and DO group, and re-revision ACL reconstruction was performed. Five of 18 patients in the SB, 5 of 15 patients in the SO, 3 of 9 patients in DB, 1 of 6 patients in DO group had decreased the level of sports activity.

## Discussion

The key finding and clinical relevance of this study are that OTTR can be used to achieve comparable results to anatomical revision surgery in cases where the bone tunnel cannot be used due to its enlargement.

Preoperative planning for revision ACLR involves the evaluation of the primary bone tunnel, selection of single or second stage, selection of the graft tendon to be used, and the method of reconstruction [2, 6, 10, 11, 18, 19, 22]. Preoperative bone tunnel evaluation can be most accurately assessed with 3D-CT, and the decision to use a bone tunnel can be made preoperatively [9]. Van Tol FR and Thomas NP reported that two-stage revision ACL reconstruction is effective for proper graft fixation. However, Van Tol showed that approximately 10% of patients developed a new meniscal tear between the first and second stage [17, 19]. Dragoo JL reported that revision ACLR utilizing a single-stage tibial tunnel grafting technique resulted in improved knee pain, function, and stability at a minimum 24-months follow-up [6]. Good results can be obtained with single-stage and second-stage surgeries, although single-stage surgery is preferable, because the meniscus and cartilage damage may progress during the waiting period in second-stage surgery, and the time until return to sporting activities is longer [19].

As for the choice of graft tendon, several methods can be used, such as using BTB, quadriceps tendon, and hamstring tendon, all of which have been reported to provide good results [11, 19, 22]. Therefore, it is considered best to use the surgeon’s preferred tendon.

OTTR procedure was reported in the 1970s by MacIntosh et al. for the first time [12], and some publications have shown various modified procedures and good clinical results [4, 16, 21]. We compared the single OTTR procedure with the single bone tunnel procedure in revision ACLR. The results were similar in terms of anterior and rotational stability by intraoperative navigation and postoperative evaluation of the Lachman test and Pivot shift test [18]. In addition, Asai et al. reported that there was no difference between the single tunnel and OTTR methods in the evaluation of rotational stability using the Pivot-shift test and electromagnetic sensor in a cadaver, and that this method may be useful in revision ACLR and pediatric primary ACLR [1]. Nagai et al. reported that primary ACLR in skeletally immature patients and revision ACLR in skeletally mature patients restored the anterior and rotational stability by using the OTTR procedure [14]. In the current study, the clinical results of single and double OTTR procedures were almost equivalent to those of the bone tunnel procedure in revision ACLR. Therefore, OTTR procedures are useful methods for cases of revision ACLR.

In terms of revision single or double bundle, Jiang et al. reported two bundles of revision ACLR with an average Lysholm score of 87.3 points and an average difference of 2.0 mm between the affected and healthy sides in tibial anterior translation using the KT-2000 at a postoperative follow-up of more than 2 years [10]. Zantop et al. reported that all patients showed less than 2.0 mm in double bundle revision ACLR at 2 years postoperatively [23], and Usman et al. reported an average difference of 0.6 mm in tibial anterior translation at 1 year postoperatively in single revision ACLR [18].

The limitation of this study is that the number of double OTTR cases is small. Since double OTTR method is only indicated when a new PL bone tunnel can be made or the initial PL bone tunnel can be used, the percentage of total revision cases for double OTTR method is limited. In our study, there was no statistically significant difference between the four surgical procedures (single bone tunnel, single OTTR, double bone tunnel and double OTTR) in manual examination results (Lachman and Pivot-shift tests) and the side-to-side difference in the tibial anterior translation. Compared to the results of previous studies, it seems that the best approach to revision ACLR is to perform with OTTR as an option, in single stage and without being concerned about creating a new tunnel or using a primary tunnel.

## Conclusion

The clinical results of single and double OTTR revision ACLR are equivalent to those of single and double bone tunnel procedures. In revision ACLR, it was thought that good results can be obtained by selecting the most appropriate method for each case in single stage.

## Abbreviations

ACLR: Anterior cruciate ligament reconstruction
OTTR: Over-the-top route
AM: Anteromedial
PL: Posterolateral
